# Phylogeography of the dark kangaroo mouse, *Microdipodops megacephalus*: cryptic lineages and dispersal routes in North America's Great Basin

**DOI:** 10.1111/j.1365-2699.2010.02472.x

**Published:** 2011-06

**Authors:** John C Hafner, Nathan S Upham

**Affiliations:** 1 Moore Laboratory of Zoology and Department of Biology, Occidental CollegeLos Angeles, CA 90041, USA; 2 Committee on Evolutionary Biology, University of ChicagoChicago, IL 60637, USA; 3 Department of Zoology, Field Museum of Natural HistoryChicago, IL 60605, USA

**Keywords:** Conservation biogeography, cryptic species, directional analysis, Great Basin, haplotype–area curves, kangaroo mice, *Microdipodops megacephalus*, mitochondrial DNA, phylogeography, source–sink dynamics

## Abstract

**Aim:**

The rodent genus *Microdipodops* (kangaroo mice) includes two sand-obligate endemics of the Great Basin Desert: *M. megacephalus* and *M. pallidus*. The dark kangaroo mouse, *M. megacephalus,* is distributed throughout the Great Basin and our principal aims were to formulate phylogenetic hypotheses for this taxon and make phylogeographical comparisons with its congener.

**Location:**

The Great Basin Desert of western North America.

**Methods:**

DNA sequence data from three mitochondrial genes were examined from 186 individuals of *M. megacephalus,* representing 47 general localities. Phylogenetic inference was used to analyse the sequence data. Directional analysis of phylogeographical patterns was used to examine haplotype sharing patterns and recover routes of gene exchange. Haplotype–area curves were constructed to evaluate the relationship between genetic variation and distributional island size for *M. megacephalus* and *M. pallidus*.

**Results:**

*Microdipodops megacephalus* is a rare desert rodent (trapping success was 2.67%). Temporal comparison of trapping data shows that kangaroo mice are becoming less abundant in the study area. The distribution has changed slightly since the 1930s but many northern populations now appear to be small, fragmented, or locally extinct. Four principal phylogroups (the Idaho isolate and the western, central and eastern clades) are evident; mean sequence divergence between phylogroups for cytochrome *b* is *c*. 8%. Data from haplotype sharing show two trends: a north–south trend and a web-shaped trend. Analyses of haplotype–area curves reveal significant positive relationships.

**Main conclusions:**

The four phylogroups of *M. megacephalus* appear to represent morphologically cryptic species; in comparison, a companion study revealed two cryptic lineages in *M. pallidus*. Estimated divergence times of the principal clades of *M. megacephalus* (*c.* 2–4 Ma) indicate that these kangaroo mice were Pleistocene invaders into the Great Basin coincident with the formation of sandy habitats. The north–south and web patterns from directional analyses reveal past routes of gene flow and provide evidence for source–sink population regulation. The web pattern was not seen in the companion study of *M. pallidus*. Significant haplotype–area curves indicate that the distributional islands are now in approximate genetic equilibrium. The patterns described here are potentially useful to conservation biologists and wildlife managers and may serve as a model for other sand-obligate organisms of the Great Basin.

## Introduction

A principal goal in conservation biology is the conservation of genetic diversity in natural populations (Frankham, 1996; Jones *et al.*, 1996; Van Dyke, 2008). Over the past two decades, the basic methods of phylogeography have proven invaluable in providing a framework for surveying genetic variation in natural populations (Avise, 2000) and phylogeographical studies have yielded much useful data for conservation biologists, evolutionary biologists, and wildlife managers. In addition to identifying patterns of genetic variation, one of the most exciting aspects of phylogeographical studies is the production of biogeographical models and the discovery of morphologically cryptic species.

With the ever-increasing loss of natural habitat in the Great Basin of western North America (Mack, 1981; Whisenant, 1990; Knapp, 1996; Pellant *et al.*, 2004; Mensing *et al.*, 2006), recent attention has focused on the conservation biology of organisms in the Great Basin, including the endemic kangaroo mice of the heteromyid rodent genus *Microdipodops* Merriam (e.g. Hafner *et al.*, 2008). Although there is a dearth of detailed information on the ecology and general natural history of these rodents, available data indicate that kangaroo mice are ecological specialists that are restricted to open, sandy habitats (Hall, 1941; Hafner *et al.*, 1996). Not surprisingly, kangaroo mice are found in some of the most arid regions of the Great Basin Desert, exhibit a patchy distribution, and are generally considered by desert naturalists to be rare (Hall, 1941; Hafner, 1981; Hafner *et al.*, 1996, 2008). It follows that a comprehensive understanding of the phylogeographical patterns for *Microdipodops* will provide the necessary footing for informed conservation management decisions and, simultaneously, provide a model for future studies of other sand-dwelling and sand-obligate organisms in the Great Basin.

Two species of kangaroo mice are currently recognized: the dark kangaroo mouse, *Microdipodops megacephalus* Merriam, and the pallid kangaroo mouse, *M. pallidus* Merriam (Patton, 2005). As indicated by their vernacular names, the present species-level taxonomy of the genus reflects a philosophy that emphasizes morphological differentiation and dates to the middle of the last century (Hall, 1941, 1946). It is now known that pelage colour varies greatly over geography in both taxa and, as such, simple darkness or paleness of the pelage is now considered an unreliable means of identifying kangaroo mice (Hafner, 1981). Indeed, discrimination of the two forms using only morphological characters is difficult and the forms are considered ‘classic sibling species’ (Hafner *et al.*, 1979, p. 8).

The remarkable phenotypic similarity of the two forms of kangaroo mice belies their evolutionary past. Although once thought to be young ‘species in the making’ (Hall, 1941; p. 237), *M. megacephalus* and *M. pallidus* are now known to be genetically isolated from each other (Hafner *et al.*, 1979) and, indeed, represent rather ancient lineages that diverged about 8 Ma (Hafner *et al.*, 2007). The recent phylogeographical study of *M. pallidus* (Hafner *et al.*, 2008) showed that this taxon was a sister clade to *M. megacephalus* and that *M. pallidus* represented two trenchant evolutionary lineages. Hence, the taxon *M. pallidus* is likely to be a complex of two morphologically cryptic species awaiting formal systematic treatment and taxonomic revision (Hafner *et al.*, 2008).

The present study treats the molecular phylogenetics and historical biogeography of *M. megacephalus*. This research is designed as a companion study to Hafner *et al.*'s (2008) analysis of *M. pallidus* and, as such, completes a phylogeographical survey of the genus. Relative to *M. pallidus*, *M. megacephalus* seems to be morphologically and ecologically less specialized (Hall, 1941; Hafner, 1981; Hafner *et al.*, 2008) and its geographical range (*c*. 180,000 km^2^) is about 4.5 times larger than that of *M. pallidus*. To facilitate comparisons with the phylogeographic patterns shown for *M. pallidus* (Hafner *et al.*, 2008), we sequenced the same three mitochondrial gene fragments used in that study to infer phylogenetic relationships in this study. Additionally, we incorporated the methodology of directional analyses introduced in Hafner *et al.* (2008) to trace historical patterns of gene exchange.

## Materials and methods

### Fieldwork and specimens examined

**Figure 1:**
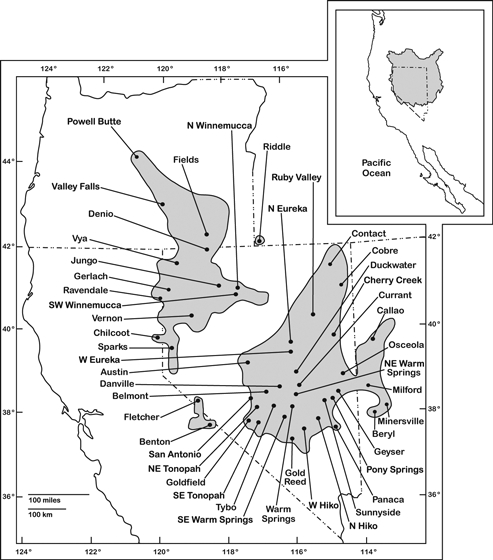
Map showing the distribution of the dark kangaroo mouse, *Microdipodops megacephalus*, and the 47 general localities sampled in this study. The inset map of western North America depicts the Great Basin Desert (shaded area) as defined on floristic data from Cronquist *et al.* (1972). In both maps, the outline of the state of Nevada is shown for orientation.

Specimens of the dark kangaroo mouse were sampled throughout its distribution in the Great Basin Desert. Of 63 specific localities sampled, localities less than *c*. 5 km apart were pooled yielding 47 localities that are hereafter referred to as general localities (Fig. 1 & Appendix S1 in Supporting Information). Specimens from a general locality were treated as a population for purposes of this study. The molecular study relied on sequence data from three mitochondrial gene fragments, 16S ribosomal RNA (16S), cytochrome *b* (cyt *b*) and transfer RNA for glutamic acid (tRNA^Glu^), and involved 186 specimens of *M. megacephalus* (Appendix S1): 172 specimens were collected between 1999 and 2007 specifically for this study, 11 specimens were collected in 1975 and 1976 in the course of a related project, and toe-clip samples were obtained from three museum specimens for analyses of ancient DNA. Mitochondrial DNA (mtDNA) sequence data from 21 specimens were taken from Hafner *et al.* (2006): GenBank accession numbers for 16S and cyt *b* are DQ422889–DQ422909 and DQ422916–DQ422936, respectively. All sequences of cyt *b* (either from GenBank or newly generated) include a small, 5′ adjoining section of tRNA^Glu^.

Outgroup taxa selected for analysis included the pallid kangaroo mouse (*M. pallidus*), the chisel-toothed kangaroo rat (*Dipodomys microps* Merriam) and the desert kangaroo rat (*D. deserti* Stephens) (Appendix S1). Outgroup samples for *M. pallidus* represented the two (eastern and western) lineages identified in Hafner *et al.* (2008). Selection of outgroup taxa was supported by previous studies (Hafner, 1982, 1993; Hafner & Hafner, 1983; Rogers, 1990; Mantooth *et al.*, 2000; Alexander & Riddle, 2005; Hafner *et al.*, 2006, 2007, 2008). Sequence data for *D. microps* were taken from Hafner *et al.* (2006): GenBank accession numbers for 16S and cyt *b* are DQ422887 and DQ422914, respectively. Sequence data for the other three outgroup specimens were taken from Hafner *et al.* (2008): GenBank accession numbers for 16S and cyt *b* for *D. deserti* are DQ870428 and DQ870429, respectively; GenBank numbers for the two individuals of *M. pallidus* are DQ534261 and DQ534357 for 16S, and DQ534255 and DQ534351 for cyt *b*, respectively. Animals collected in this study were treated in a humane manner following guidelines of the American Society of Mammalogists (Gannon *et al.*, 2007) and Occidental College's Institutional Animal Care and Use Committee.

### Analyses of mtDNA

All laboratory procedures related to DNA extraction from freshly frozen tissues, including mtDNA amplification using polymerase chain reaction (PCR), purification and sequencing, were conducted as described in Hafner *et al.* (2008). Following Hafner *et al.* (2008), two principal mitochondrial genes, 16S and cyt *b*, were analysed because they have contrasting nucleotide substitution rates (16S evolves more slowly than cyt *b*; Ferris *et al.*, 1983; Springer *et al.*, 2001; Hafner *et al.*, 2006, 2007). It should be noted that sequencing of the cyt *b* gene yielded a continuous section of a small (40 base pairs, bp) portion of tRNA^Glu^, five non-coding bases, and 403 bp of the protein-coding cyt *b* gene (Hafner *et al.*, 2008). As in Hafner *et al.* (2008), the continuous section of tRNA^Glu^ and cyt *b* was not involved in separate cyt *b* analyses but used only in the phylogenetic analysis of the combined data set (16S + cyt *b*+ tRNA^Glu^).

Ancient mtDNA analyses were used to obtain genomic DNA from three museum specimens: SDNHM 16431, IMNH 259 and IMNH 693 (collected in 1920, 1968 and 1977, respectively). The lateral digit of the right manus was removed from each specimen and cut into small pieces so that a combination of dried tissue, bone, hair and nail (*c*. 2 mm^2^) was the starting material for DNA extraction. DNA was extracted using the DNeasy Blood & Tissue Kit (QIAGEN Inc., Valencia, CA, USA) following the modifications of Iudica *et al.* (2001) to the manufacturer's protocol. Several steps were altered to improve DNA yield: tissue samples were soaked in phosphate-buffered saline for 24 h prior to digestion (with three to five solution changes), digested for 48–72 h at 55 °C until fully homogenized, and carrier nucleotides were added prior to the final elution to facilitate DNA precipitation (3 μg of yeast tRNA; Hafner *et al.*, 2005; J.W. Demastes, University of Northern Iowa, Cedar Falls, IA, USA, pers. comm.). The fragmented nature of the extracted DNA necessitated the use of internal primers to obtain the full length of the targeted 16S and cyt *b* gene fragments (542 bp and 448 bp, respectively). Internal primers were designed specifically for *M. megacephalus* to amplify short (300 bp or less), overlapping segments of each gene that could then be assembled to reach the desired total length. Primers designed for this study are listed in Appendix S2, along with the primer pair combinations used for PCR amplification and sequencing. PCR amplifications were performed in 25 μL reaction volumes using 12.5 μL (0.75 U) of JumpStart REDTaq Ready Mix (Sigma, St. Louis, MO, USA), 10.5 μL of sterile water, 0.5 μL of each primer (10 μm), and 1.0 μL of template DNA. The thermal profile for amplifications of ancient mtDNA included one initial cycle at 95 °C (2 min), followed by 35 cycles of denaturation at 95 °C (30 s), annealing at 55 °C (30 s), extension at 72 °C (30 s), and a final extension at 72 °C (10 min). Purification of PCR products and direct sequencing were performed as described by Hafner *et al.* (2006).

Precautions were taken to address contamination concerns associated with analyses of ancient DNA. Prior to laboratory work, bench surfaces and equipment were washed with a DNase solution (DNA Away, Molecular Bio-Products, San Diego, CA, USA) to remove DNA. Procedures pertaining to DNA extraction and amplification were performed in a separate area of the laboratory using dedicated pipettors with Aerosol Resistant Tips (ART; Molecular Bio-Products). All PCRs were run with two negative controls to detect contamination. Since many of the primers were designed in *Microdipodops*-specific or *M. megacephalus*-specific regions, the chance of amplifying an incorrect gene target was reduced. Nonetheless, the identity of all mtDNA gene sequences was verified using BLAST (Basic Local Alignment Search Tool, National Center for Biotechnology Information, Bethesda, MD, USA). All ancient mtDNA sequences were compared to sequences from unrelated laboratory activity to ensure that each sequence was the product of amplification from the target template. Following Pääbo *et al.* (2004), results were verified by obtaining multiple DNA extractions from each specimen and performing multiple independent amplifications on each DNA extract.

### Phylogenetic analyses

Sequences pertaining to the light and heavy strands for each individual were edited and assembled following the methods of Hafner *et al.* (2008). New sequences pertaining to individuals of *M. megacephalus* (*n*=163) were submitted to GenBank (GenBank accession numbers DQ870226–DQ870280, DQ870282–DQ870312, DQ870314–DQ870326, and EU861064–EU861127 for 16S; DQ870327–DQ870361, DQ870363–DQ870404, DQ870406–DQ870427, and EU861128–EU861191 for cyt *b*). Alignment of multiple sequences and examination and editing of alignments to verify gap placement were performed as described in Hafner *et al.* (2008). Although the alignment of 16S sequences was unambiguous between *M. megacephalus* individuals and the *M. pallidus* outgroup taxa, secondary structural models were consulted to resolve ambiguous gap regions in the 16S alignment of *M. megacephalus* with the two *Dipodomys* outgroups. Note that the 16S and combined data sets in this study are 1 bp shorter than in Hafner *et al.* (2008) due to the correction in the 16S alignment pertaining to a false gap (correction of a false autapomorphy in the *D. deserti* outgroup sequence). Unresolved ambiguous sites in the complete 16S alignment were treated independently in the subsequent phylogenetic analyses; their inclusion or exclusion caused only minor changes in the extreme terminal tree branches, so ambiguous sites were retained to improve the phylogenetic resolution in the analyses of the 16S and combined data sets. Unique haplotypes were identified using MacClade 4.0 (Maddison & Maddison, 2000) and all phylogenetic analyses were based on unique haplotypes. Methods for determining transition/transversion ratios, estimating base composition, and testing for saturation followed Hafner *et al.* (2008).

Sequence variation in the protein-coding cyt *b* gene was tested for the influence of natural selection and possible deviations from selective neutrality using the McDonald–Kreitman test (McDonald & Kreitman, 1991) performed in DnaSP 5.00.07 (Librado & Rozas, 2009). For this test, unique haplotypes of *M. megacephalus* were identified as the focal group and all unique haplotypes of *M. pallidus* (Hafner *et al.*, 2008) as the outgroup. Data from the non-coding 16S gene were presumed to meet the assumption of selective neutrality.

To investigate possible incongruence between the gene fragments (Wiens, 1998; Leaché & Reeder, 2002), phylogenetic analyses were first performed separately on the 16S (542 bp) and cyt *b* (403 bp) data sets, then on the combined (16S + cyt *b*+ tRNA^Glu^) alignment of 990 bp. The partition homogeneity test (PHT; Farris *et al.*, 1994) was conducted as in Hafner *et al.* (2008) using paup* 4.0b10 (Swofford, 2003) to further evaluate phylogenetic congruence. A non-significant PHT result (*P*=0.98) permitted combination of the three mtDNA gene fragments. Phylogenetic analyses of the three mtDNA data sets (16S, cyt *b* and combined) using maximum-parsimony and neighbour-joining methods (paup* 4.0b10) determined that all trees were virtually identical topologically except for minor changes within the terminal branches. The combined data set was also analysed using Bayesian (MrBayes 2.01; Huelsenbeck & Ronquist, 2001) and maximum-likelihood (RAxML 7.0.4; Stamatakis, 2006) methods.

Maximum-parsimony analyses followed the methods of Hafner *et al.* (2008). Nodal support for the consensus tree was evaluated using 1000 bootstrap pseudoreplicates (Felsenstein, 1985) and Bremer support values (Bremer, 1994) were obtained by using paup* 4.0b10 and TreeRot 2 (Sorenson, 1999). Tests for presence of phylogenetic signal (Hillis & Huelsenbeck, 1992) and calculations of the consistency index (CI) and retention index (RI) were conducted using paup* 4.0b10.

Measures of genetic distances were calculated to facilitate direct comparison with results from Hafner *et al.* (2008). mega 3.1 (Kumar *et al.*, 2004) was used to estimate percentage nucleotide sequence divergence using both uncorrected pairwise (*p*) distance and Kimura's two-parameter model (Kimura, 1980). Following the methods of Hafner *et al.* (2008), neighbour-joining distance trees (Nei & Kumar, 2000) were constructed using uncorrected *p* distance.

Determination of the most suitable model of nucleotide evolution for the combined data set was made using Modeltest 3.7 (Posada & Crandall, 1998) under the Akaike information criterion (AIC) (Posada & Buckley, 2004). The transversional model with invariant sites and among-site rate variation (TVM + *I*+ Γ) was identified as the most appropriate model. Bayesian phylogenetic analyses were performed as in Hafner *et al.* (2008) with MrBayes 2.01 but with two modifications. First, the program was executed using the GTR + *I*+ Γ model (TVM not available) with parameters estimated under uniform priors by each Bayesian analysis (Leaché & Reeder, 2002). Second, incrementally heated chains (Metropolis-coupled Markov chain Monte Carlo; Huelsenbeck & Ronquist, 2001) were run and sampled following Hafner *et al.* (2008) but the first 2000 trees prior to stationarity were conservatively eliminated for each of two runs as burn-in values. The remaining 16,000 equilibrium trees combined from both analyses were used to calculate nodal posterior probabilities and to create a 50% majority-rule consensus tree.

Maximum-likelihood analyses were conducted using RAxML due to the large number of taxa in the combined data set and extended computational times. The rapid algorithms of RAxML are optimized using the general time-reversible (GTR+ Γ) model of rate heterogeneity (Stamatakis, 2006). Thus maximum-likelihood and bootstrap searches were performed under this model, partitioning by gene fragment (the GTR model differs from the TVM model by estimating six rate parameters rather than five). The run was repeated several times with random starting trees to verify topology, and clade support was assessed using 1000 bootstrap replicates. Due to difficulties resolving outgroup placement, and the presumed reciprocal monophyly of the ingroup and outgroups, subsequent runs used the −g option in RAxML to constrain the monophyly of *M. pallidus* relative to *M. megacephalus*. The resulting best-scoring maximum-likelihood tree was annotated with support values from bootstrap replicate trees.

### Divergence-time analyses

Adherence to a global molecular clock model was evaluated using a log-likelihood ratio test between clock-constrained and non-constrained trees, as implemented in paup* 4.0b10 under the TVM + *I*+ Γ model with fixed parameters. Clock-like rates of evolution were not rejected across the combined data set (*P*>0.05 for *Dipodomys* outgroups *+ Microdipodops* taxa, *Microdipodops* taxa only, and *M. megacephalus* only); thus, the use of strict clock and relaxed clock models was compared in the subsequent analyses. Rates either conformed to a strict molecular clock (CLOC) or were set to uncorrelated lognormal (UCLN), where rates for each branch are independently drawn from a lognormal distribution (Drummond *et al.*, 2006).

Divergence times of major clades were estimated using beast 1.5.4 (Drummond & Rambaut, 2007). Calibration priors used two independent strategies. First, the root divergence between *Dipodomys* and *Microdipodops* was calibrated to correspond with the minimum age of the oldest fossil *Dipodomys* (Reeder, 1956) that dates from the Barstovian North American Land Mammal ‘Age’ (15.9–12.5 Ma; Prothero, 1998); the root height was set to a lognormal prior distribution with an offset of 12.5 Ma, mean of 0, and standard deviation of 1. Second, two dates (and credibility intervals) were used from the Hafner *et al.* (2007) parametric Bayesian analysis of the Heteromyidae: 15.35 Ma (14.10, 15.88) for the root divergence, and 8.06 Ma (6.34, 10.01) for the divergence between *M. pallidus* and the *M. megacephalus* ingroups. These calibrations were set using normal prior distributions with mean of 15.35 Ma (standard deviation of 0.75) and mean of 8.06 (standard deviation of 1.2), respectively. The two calibration strategies using CLOC and UCLN yielded four sets of divergence-time estimates.

beast analyses were run under the TVM + *I*+ Γ model by initially selecting GTR and altering the xml file to include equal transition rates. Yule priors were selected due to the species-level scale of analysis, and reciprocal monophyly of the ingroup and outgroup was assumed a priori in accord with results from the MrBayes and paup* 4.0b10 analyses. Chain lengths were set to 10,000,000 generations with parameters sampled every 1000 generations. Two independent runs of the UCLN analyses were combined in order to converge upon stable posterior parameter distributions, as determined by Tracer 1.5 (Rambaut & Drummond, 2007); otherwise, single runs were sufficient for the CLOC analyses. Trees were summarized as maximum clade credibility trees after discarding the first 20% of each run as burn-in using the TreeAnnotator program in beast. The resulting trees contained mean divergence times and error bars for each node reporting 95% highest posterior density (HPD) intervals.

### Orientation analyses of haplotype sharing patterns

Historical trends in gene exchange of kangaroo mice were assessed using directional analyses of phylogeographical patterns, DAPP (Hafner *et al.*, 2008). DAPP relies on compass orientations between pairs of localities whose individuals share haplotypes. Axial data (angular measurements of undirected lines) were measured between all combinations of pairwise localities involved in haplotype sharing and a mean vector (μ) was calculated for each major geographical unit of *M. megacephalus*. Rayleigh's uniformity test, Rao's spacing test and Kuiper's test (Batschelet, 1981; Fisher, 1993; Kovach, 2006) were used to determine if each sample of orientations between pairwise localities was distributed isotropically. The Mardia–Watson–Wheeler test and the Watson *U*^2^ test were used to test the equality of two angular distributions. Circular statistics involved in DAPP used Oriana 2 software (Kovach, 2006).

### Haplotype sampling, diversity and distributional islands

The genetically defined, geographical units of *Microdipodops* identified in this study and in Hafner *et al.* (2008) represent mainland islands and were examined biogeographically in the context of haplotypic diversity and island size. This novel approach was inspired by empirical observations regarding population size and genetic variation (Soulé, 1976; Frankham, 1996) and the theory of island biogeography (MacArthur & Wilson, 1967; MacArthur, 1972). Areas (km^2^) of distributional islands of kangaroo mice were obtained using VistaMetrix 1.35 software (SkillCrest, LLC, Tucson, AZ, USA) that provided a transparent overlay for recording areas from underlying distribution maps; following convention (e.g. MacArthur, 1972; Frankham, 1996), distributional island areas were converted to log values before analysis. Correlation and regression analyses were used to evaluate hypothesized relationships between the number of unique composite haplotypes, *h*, and the log of the distributional island area. Haplotype–area curves were evaluated separately for the species of kangaroo mice and across all distributional islands for the genus. Estimation of the completeness of haplotype sampling was made following Dixon (2006); for each distributional island, the probability of completeness, *P* (the probability that all haplotypes were sampled), and the predicted number of haplotypes, *ĥ*, were calculated. Statistical routines were performed using systat 11 (SYSTAT Software, Inc., 2004).

## Results

### Fieldwork and geographical distribution

Fieldwork, involving the capture of 199 individuals of *M. megacephalus* from 27,014 trapnights, yielded an overall trapping success of 0.74% for *M. megacephalus*. Although traps were set at known localities (Hall, 1941; Hafner, 1981) or at new sites in habitats judged (by J.C.H.) to be appropriate for this species, trapping success was only 2.67% when considering only those localities where individuals of *M. megacephalus* were captured. The range in trapping success was 0.25% (one capture from 400 trapnights) to 18.0% (9 captures from 50 trapnights) at localities that yielded *M. megacephalus*.

Our understanding of the present geographical distribution of *M. megacephalus* (Fig. 1) is similar to Hall's (1941) description but with several notable differences. Field collection since Hall's (1941) study has yielded two main distributional adjustments: (1) the presence of a distributional isolate in Idaho (Hafner, 1985); and (2) a range extension into the Escalante Desert of south-western Utah (i.e. the localities of Minersville and Beryl reported in this paper). Each of these distributional adjustments extends the known range of *M. megacephalus* more than 100 km from other known populations of the species. In addition, fieldwork and examination of museum specimens revealed that the kangaroo mice around the southern end of Pyramid Lake (western Nevada) are not *M. megacephalus* (cf. Hall, 1941, 1946; Mantooth *et al.*, 2000) but are *M. pallidus* (see Hafner *et al.*, 2008). The distribution of *M. megacephalus* in this region is therefore restricted (generally to the north and to the west of Pyramid Lake) relative to that described in Hall (1941).

### Sequence characteristics

Analysis of the combined (16S + cyt *b*+ tRNA^Glu^) sequence shows 242 variable characters (99, 136 and 7 variable characters, respectively) across all unique haplotypes of *M. megacephalus* and outgroup taxa. Mean base frequencies for A, C, G and T across all samples are 0.313, 0.245, 0.166 and 0.277, respectively (0.330, 0.209, 0.193 and 0.268, respectively, for 16S; and 0.279, 0.294, 0.138 and 0.288, respectively, for cyt *b*; data for tRNA^Glu^ available on request from J.C.H.). Chi-square tests for possible heterogeneity of base frequencies across all samples are not significant for the combined data set (χ^2^=11.602, *P*=1.000) or for each gene (χ^2^=8.272, *P*=1.000 for 16S; χ^2^=14.940, *P*=1.000 for cyt *b*); hence, it is unlikely that base compositional heterogeneity causes phylogenetic bias. Mean base frequencies for A, C, G and T for unique *M. megacephalus* haplotypes only are 0.330, 0.209, 0.192 and 0.269, respectively, for 16S and 0.279, 0.294, 0.138 and 0.289, respectively, for cyt *b*.

Investigation of the possible role of natural selection in sculpting sequence variation in protein-coding cyt *b* reveals selective neutrality. All fixed substitutions between *M. megacephalus* and *M. pallidus* are due to synonymous substitutions (the analysis includes 64 unique haplotypes for *M. megacephalus* from this study and 26 unique haplotypes for *M. pallidus* from Hafner *et al.*, 2008). The results of the McDonald–Kreitman test (McDonald & Kreitman, 1991) for selective neutrality of sequence variation in cyt *b* show that the ratio of the number of non-synonymous (0) to synonymous (9) fixed substitutions between *M. megacephalus* and *M. pallidus* is not significantly different from the ratio of non-synonymous (11) to synonymous (110) polymorphisms within the species (Fisher's exact test, *P*=0.608).

Plots of the number of transitions versus uncorrected *p* distance (following the methods of Barker & Lanyon, 2000), show no evidence for saturation for 16S or for cyt *b* for the unique haplotypes of *Microdipodops* studied. Saturation is seen in third-position transitions for cyt *b* when *D. deserti* and *D. microps* are included in the analyses. Transition/transversion ratios for 16S, cyt *b* and the combined data set are 1.865, 8.447 and 4.256, respectively, for samples of *M. megacephalus* only (over all positions and using uncorrected *p*). Tests for phylogenetic signal in our data (involving all unique haplotypes and species of *Dipodomys* designated as outgroups) show significance for 16S (99 variable characters, 50 haplotypes, skewness, g_1_, = −0.478, *P*<0.01) and for cyt *b* (136 variable characters, 68 haplotypes, g_1_=−0.370, *P*<0.01).

### Mitochondrial DNA variation in *Microdipodops megacephalus*

Analysis of the combined mtDNA data set for *M. megacephalus* (including 186 individuals from 47 general localities) reveals 88 unique composite haplotypes and 141 polymorphic sites. Examining 16S and cyt *b* separately, there are 46 and 64 unique haplotypes and 50 and 91 polymorphic sites for these genes, respectively.

An assessment of intrapopulational mitochondrial sequence variation may be made by examining the 38 general localities represented by multiple individuals. There is a mean of 4.66 (range 2–21) individuals sampled per locality for these 38 localities. There are significant functional relationships between the number of haplotypes and sample size seen at a locality for 16S (*b*=0.085, *P*=0.034), cyt *b* (*b*=0.230, *P*=0.000), and for composite haplotypes (*b*=0.257, *P*<0.001). In all comparisons, measures of within-population variation are lower for 16S than for cyt *b*. For example, the mean number of haplotypes per locality is 2.05 and 2.50 for 16S and cyt *b*, respectively. Additionally, the mean number of polymorphic sites per population is 2.05 and 3.89 for 16S and cyt *b*, respectively.

### Phylogenetic patterns

**Figure 2:**
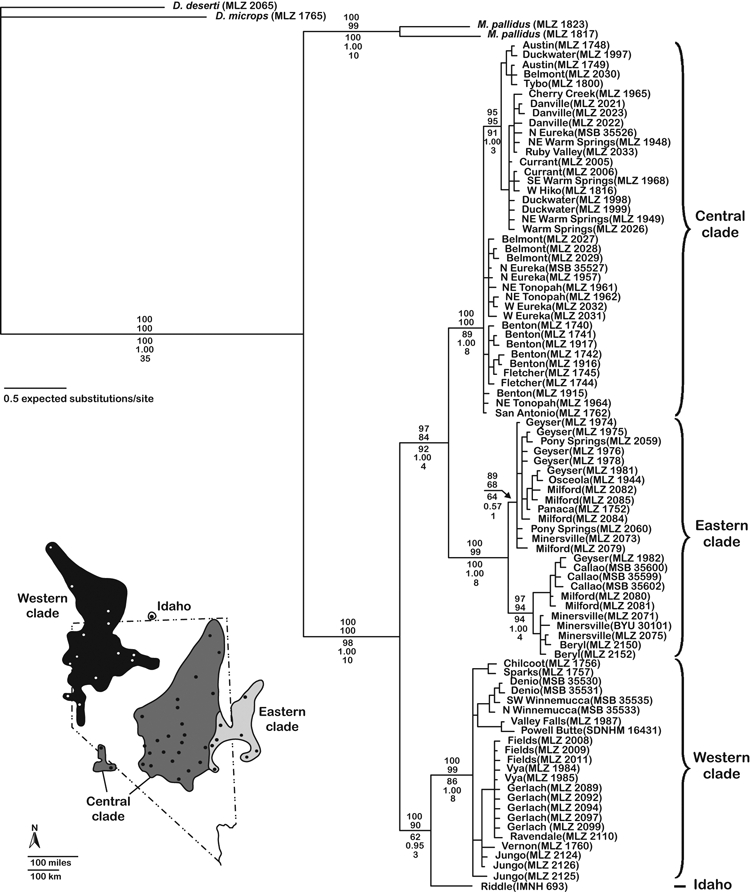
Bayesian phylogenetic tree based on the composite mtDNA sequence data and showing the relationships among the 88 unique haplotypes of *Microdipodops megacephalus* from the Great Basin Desert region of western North America. Distance and parsimony bootstrap support values are indicated above the nodes, with maximum-likelihood support values, Bayesian posterior probabilities and Bremer decay indices below the nodes. The inset map at the lower left shows the geographic range of the four principal clades.

Phylogenetic analysis of the combined (990 bp) mtDNA data for the 88 ingroup haplotypes of *M. megacephalus* and the four outgroup species yields 174 characters that are potentially parsimony informative (70, 98 and three parsimony-informative characters for the separate 16S, cyt *b* and tRNA^Glu^, respectively). Maximum-parsimony analysis of the data set shows over 10,000 most-parsimonious trees (topologies are the same for 500 of 10,000 trees; CI = 0.732; RI = 0.910). Analyses using maximum-parsimony, neighbour-joining, maximum-likelihood and Bayesian approaches yield trees having virtually identical topology and differing only in the placement of *M. megacephalus* haplotypes at extreme terminal branches. As was seen in Hafner *et al.* (2008), monophyly of the genus *Microdipodops* is strongly supported in all analyses. All analyses except the unconstrained maximum-likelihood approach show that the 88 unique haplotypes of *M. megacephalus* form a highly resolved sister clade relative to the samples of *M. pallidus* (Fig. 2). It appears that without the ingroup monophyly constraint, the maximum-likelihood method suffers from the taxon-number imbalance between ingroup and outgroup, and becomes trapped too early in a local optimum.

Four major phylogroups are recognized with high resolution within *M. megacephalus* (Fig. 2): central clade, eastern clade, western clade and the peripheral isolate from Idaho (the Riddle locality). These four clades comprise two sister lineages that assort into a basal south-eastern unit (including the central and the eastern clades) and a basal north-western unit (including the western clade and the Idaho isolate that is known from only one general locality). The four phylogroups appear to be distributed entirely in an allopatric fashion.

The central clade (Fig. 2) consists of one well-resolved subclade and a poorly resolved assemblage of unique haplotypes. The subclade consists of 20 unique haplotypes and represents 21 of the 25 localities of the central clade (excluding the localities of W Eureka, San Antonio, Fletcher and Benton; Figs 1 & 2). The remaining assemblage of 19 unique haplotypes shows little structure in the parsimony, maximum-likelihood and Bayesian analyses but is recognized as a sister subclade in the neighbour-joining analysis (bootstrap support of 83). Unlike the well-resolved subclade, this assemblage is distributed narrowly (includes only Fletcher, Benton, San Antonio, NE Tonopah, Belmont, W Eureka and N Eureka) in the western portion of the geographic range of the central clade (Figs 1 & 2). Nearly half (eight of 19) of the unique haplotypes in this assemblage are contributed by kangaroo mice inhabiting the Mono Basin region of California and Nevada (localities of Fletcher and Benton; Figs 1 & 2). Haplotypes belonging to the well-resolved subclade and the assemblage co-occur at three western localities: NE Tonopah, Belmont, and N Eureka.

Haplotypes of the eastern clade assort into two well-resolved phylogeographical subunits: a western subunit (distributed mainly to the west of the Nevada State boundary) and an eastern subunit (distributed primarily east of the Nevada boundary in the State of Utah; Figs 1 & 2). The western subunit consists of 14 unique haplotypes and its distribution includes the localities of Panaca, Pony Springs, Geyser, Osceola, Milford and Minersville. The eastern subunit consists of 11 unique haplotypes and includes the localities of Beryl, Minersville, Milford, Callao and Geyser. Admixing of subunit haplotypes is seen at the central localities of Geyser (eight of 10 individuals have the western haplotype), Milford (six of 10 individuals show the western haplotype), and Minersville (one of 10 individuals shows the western haplotype; Figs 1 & 2).

The western clade includes a rather heterogeneous collection of 23 unique composite haplotypes from 13 localities. Relationships among the haplotypes within the western clade are resolved poorly (Fig. 2). The western clade is best viewed as a complex polytomy and, as such, lacks the structure seen in the central and eastern clades. Lastly, the Idaho isolate (the Riddle locality) is represented by one haplotype and is aligned in a sister-group fashion with the western clade.

Although levels of sequence divergence within the major clades of *M. megacephalus* are moderate (*c*. 1.5–2.1% for cyt *b*; Table 1), the principal clades are recognized by high levels of sequence divergence (*c*. 5.5–10.2% for cyt *b*; Table 1). Among the inter-clade comparisons, the smallest divergence values are seen in the contrast between the western clade and the Idaho isolate. The largest divergence values are recorded between the western and the eastern clades (Table 1). Due to the known higher rate of nucleotide substitution of cyt *b*, sequence divergence values both within and between *Microdipodops* clades are consistently greater for cyt *b* than corresponding values for 16S (Table 1).

**Table 1:** Mean pairwise sequence-divergence values within and among selected clades of *Microdipodops* from the Great Basin Desert region of western North America examined in this study. Mean percentage divergence estimates for both uncorrected pairwise (*p*) distance and Kimura's two-parameter model (in parentheses) are given for individual genes and the combined data set (All)

	% divergence		
	
Comparison	16S	cyt *b*	All
*Microdipodops megacephalus* contrasts			
Within western clade	0.59 (0.59)	2.08 (2.12)	1.00 (1.01)
Within central clade	0.60 (0.60)	1.49 (1.51)	0.78 (0.78)
Within eastern clade	1.00 (1.01)	1.56 (1.59)	1.09 (1.10)
Western clade versus central clade	3.61 (3.71)	8.45 (9.15)	5.33 (5.57)
Western clade versus eastern clade	4.34 (4.49)	9.32 (10.23)	6.10 (6.43)
Western clade versus Idaho isolate	1.98 (2.02)	5.28 (5.52)	3.28 (3.37)
Idaho isolate versus central clade	3.02 (3.09)	8.72 (9.47)	5.15 (5.37)
Idaho isolate versus eastern clade	3.52 (3.61)	8.75 (9.55)	5.54 (5.81)
Central clade versus eastern clade	2.68 (2.73)	6.84 (7.31)	4.16 (4.31)
*M*. *megacephalus* versus *M*. *pallidus*	6.21 (6.50)	13.39 (15.08)	9.03 (9.69)

### Estimates of divergence dates

Results of the beast analyses conforming to the molecular clock and using a relaxed clock are similar but with the latter estimates being generally older (Table 2). Of the two calibration strategies employed (i.e. the use of the single fossil date or the two dates from Hafner *et al.*, 2007), the use of the single fossil calibration yields nodal dates that are younger for the strict clock analyses but sometimes older for the relaxed clock analyses (Table 2). Across all beast analyses, estimated dates of basal divergence within *M. megacephalus* vary from *c*. 4 to 9 Ma and divergence-time estimates for the principal clades range from *c*. 2 to 7 Ma (Table 2).

**Table 2:** Estimates of divergence time for major nodes of the *Microdipodops megacephalus* phylogeny obtained from beast analyses using rates of evolution that either conformed to a strict molecular clock, CLOC, or a relaxed clock with uncorrelated lognormal rates, UCLN. Calibration priors relied on two strategies (see text): Fossil (a single fossil date at the root) and two dates estimated by Hafner *et al.* (2007). Values shown are the mean and 95% highest posterior density (HPD) interval from the maximum clade credibility tree in millions of years ago (Ma). Specimens are from the Great Basin Desert region of western North America

	Divergence time (Ma)			
	
	CLOC		UCLN	
		
Node	Fossil	Hafner *et al.* (2007)	Fossil	Hafner *et al.* (2007)
*Dipodomys*/*Microdipodops*	13.69 (12.52, 16.00)	15.26 (13.81, 16.65)	13.68 (12.53, 15.93)	14.95 (13.50, 16.44)
*M*. *pallidus*/*M*. *megacephalus*	7.05 (5.23, 8.89)	7.85 (6.50, 9.34)	11.38 (6.97, 14.71)	9.12 (7.16, 11.10)
Within *M*. *megacephalus*				
Central + Eastern/Western + Idaho	3.88 (2.79, 5.08)	4.31 (3.27, 5.39)	9.20 (5.64, 12.86)	7.78 (5.54, 10.10)
Central/Eastern	2.54 (1.74, 3.39)	2.83 (2.01, 3.64)	6.85 (3.91, 9.88)	5.99 (3.89, 8.15)
Western/Idaho	2.18 (1.42, 2.98)	2.41 (1.68, 3.24)	6.51 (3.26, 9.82)	5.64 (3.37, 8.06)

### Private haplotypes, haplotype sharing and directional analyses

**Figure 3:**
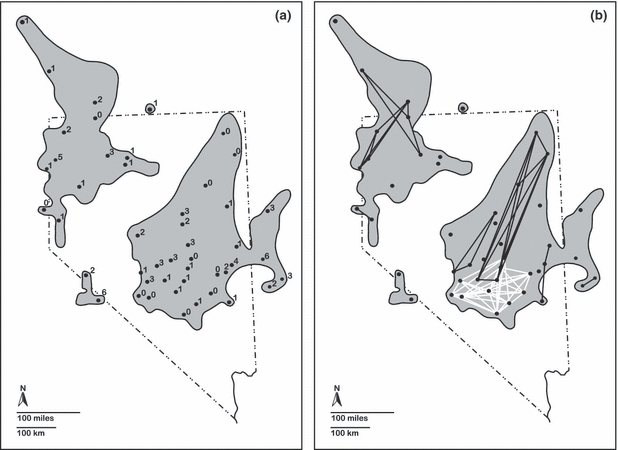
Distribution and abundance of private haplotypes (a) and pairwise haplotype sharing patterns (b) for localities of *Microdipodops megacephalus* from the Great Basin Desert region of western North America. Seventy-six of 88 unique composite haplotypes identified in this study are private haplotypes with 0–6 private haplotypes per locality (a). The remaining 12 unique composite haplotypes are present at two or more localities and yield 66 pairwise combinations of axial data of haplotype sharing (b). Two significantly different angular patterns, a north–south bi-directional trend (black lines) and a complex web pattern (white lines), are evident (b). Note that lower numbers (i.e. 0 or 1) of private haplotypes are found generally in the northern and southern portions of the distribution (a). Data pertaining to private haplotypes and haplotype sharing suggest evidence of source–sink metapopulation dynamics (see text).

Private haplotypes (restricted to only one locality) commonly occur in *M. megacephalus*. Of the 88 unique composite haplotypes identified in Fig. 2, 76 (86.4%) are private haplotypes and the remaining 12 (13.6%) are shared between and among two or more localities (Fig. 3; Table 3). The number of private haplotypes per locality varies greatly over geography (range is 0–6; Fig. 3a). In general, it appears that higher numbers of private haplotypes are recorded in the middle latitudes of the distributional ranges of the major clades and this pattern is independent of sample size. Considering those general localities with multiple individuals, there is no functional relationship between the number of private haplotypes and sample size in the central clade (*b*=0.431, *P*=0.088) and in the eastern clade (*b*=0.318, *P*=0.062). There is, however, a significant functional trend between number of private haplotypes and sample size in the western clade (*b*=0.204, *P*=0.005) but this significance is due to a single locality with large leverage, Gerlach (5 private haplotypes recorded from 21 individuals); the functional trend disappears entirely (*b*=0.120, *P*=0.320) with the removal of this one locality.

**Table 3:** Sharing of unique composite haplotypes of *Microdipodops megacephalus* from the Great Basin Desert region of western North America over geography. Twelve unique haplotypes, identified in Fig. 2, are present at two or more general localities and are available for directional analyses of phylogeographical patterns (see text). In total, there are 66 pairwise combinations of shared haplotypes (11 in the western clade, 52 in the central clade, and three in the eastern clade) that provide the basis for directional data

Unique haplotype	Number of localities	Distribution
NE Warm Springs MLZ 1949	7	Central clade: NE Warm Springs (MLZ 1949), Sunnyside (MLZ 1966), Warm Springs (MLZ 2024), SE Warm Springs (MLZ 1972), N Hiko (MLZ 1960), SE Tonopah (MLZ 1831) and Gold Reed (MLZ 2055-2058)
Currant MLZ 2006	6	Central clade: Currant (MLZ 2006), NE Tonopah MLZ (1963), SE Warm Springs (MLZ 1969-1971), Goldfield (MLZ 1747), W Hiko (MLZ 1815) and Gold Reed (MLZ 2053 and MLZ 2054)
Ruby Valley MLZ 2033	5	Central clade: Ruby Valley (MLZ 2033), Contact (MLZ 2069 and MLZ 2070), Cobre (MLZ 2067), Tybo (MLZ 1799) and Warm Springs (MLZ 2025)
Fields MLZ 2009	4	Western clade: Fields (MLZ 2009), Vya (MLZ 1986), Gerlach (MLZ 2091, MLZ 2093, MLZ 2096, MLZ 2098, MLZ 2101, MLZ 2105, MLZ 2108, and MLZ 2109) and Ravendale (MLZ 2111, MLZ 2113 and MLZ 2114)
Belmont MLZ 2028	3	Central clade: Belmont (MLZ 2028), N Eureka (MLZ 1956) and San Antonio (MLZ 1761)
Currant MLZ 2005	3	Central clade: Currant (MLZ 2005), Cobre (MLZ 2068) and NE Warm Springs (MLZ 1905 and MLZ 1950)
Denio MSB 35530	3	Western clade: Denio (MSB 35530), Valley Falls (MLZ 1993) and Jungo (MLZ 2128)
Geyser MLZ 1974	2	Eastern clade: Geyser (MLZ 1974) and Osceola (MLZ 1942 and MLZ 1943)
Geyser MLZ 1976	2	Eastern clade: Geyser (MLZ 1976 and MLZ 1979) and Panaca (MLZ 1755)
Chilcoot MLZ 1756	2	Western clade: Chilcoot (MLZ 1756 and MVZ 158930) and Sparks (MLZ 1759)
Denio MSB 35531	2	Western clade: Denio (MSB 35531) and Fields (MLZ 2007, MLZ 2010 and MLZ 2015)
Minersville MLZ 2075	2	Eastern clade: Minersville (MLZ 2075, MLZ 2077 and MLZ 2078) and Beryl (MLZ 2145-2149 and MLZ 2151)

The 12 composite haplotypes shared between and among localities yield a total of 66 pairwise combinations of axial data that are available for DAPP analysis (Table 3; Fig. 3b). Most of the haplotype sharing occurs in the central clade (52 pairwise combinations), with fewer instances of sharing in the other major clades (11 and 3 pairwise combinations in the western and eastern clades, respectively). There is no sharing of haplotypes among the major clades of *M. megacephalus* and there is no sharing of haplotypes between the Mono peripheral isolate and the main body of the central phylogroup. Visual representation of the orientation data from the DAPP (Fig. 3b) shows two distinct patterns among the *M. megacephalus* distributional bodies. One orientation pattern, involving 10 haplotypes (individual haplotype sharing from two to five localities each and a total of 30 pairwise combinations of axial data; Table 3), shows a distinct north–south directional pattern (black lines in Fig. 3b). The mean vector of the north–south pattern is μ = 23.077° [and also 203.077° because of the bi-directional (axial) nature of the data] and this pattern is found to be significantly different from a uniform distribution (Rayleigh's *Z*=10.538, *P*<0.001; Rao's *U*=160, *P*<0.05; Kuiper's *V*=2.712, *P*<0.01). The other orientation pattern is derived from the remaining two haplotypes (involving sharing among six and seven localities each and includes 36 pairwise combinations of orientation data; Table 3) and appears like a giant web in the southern portion of the distribution (white lines in Fig. 3b). These data show no departure from a uniform distribution (Rayleigh's *Z*=1.366, *P*=0.257; Rao's *U*=112, *P* > 0.90; Kuiper's *V*=1.239, *P*>0.15). Moreover, the north–south orientation trend and the web pattern (Fig. 3b) have significantly different angular distributions (Mardia–Watson–Wheeler *W*=14.72, *P*< 0.001, Watson *U*^2^=0.402, *P*<0.001).

### Genetic variation and distributional island size: haplotype–area curves

**Figure 4:**
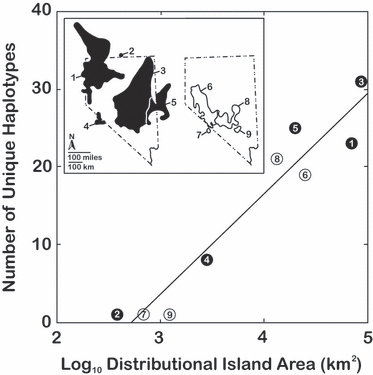
Haplotype–area curve for the distributional islands of *Microdipodops* from the Great Basin Desert region of western North America. Distributional islands for *M. megacephalus* (closed circles; shaded inset map on left): (1) western clade; (2) Idaho isolate; (3) main central unit; (4) Mono isolate; and, (5) eastern clade. Distributional islands for *M. pallidus* (open circles; unshaded inset map on right): (6) main western unit; (7) Deep Springs isolate; (8) main eastern unit; and, (9) Alamo isolate. Highly significant functional relationships exist between the number of observed unique composite haplotypes and area for the distributional islands of kangaroo mice, regardless of whether the curves are evaluated separately for the taxa or combined for the nine distributional islands (as shown, *b*=12.918, *P*<0.001; *r*^2^=0.922). The significant haplotype–area curves suggest that the populations of kangaroo mice represented by the distributional islands are now in approximate genetic equilibrium (see text and Table 4).

Five distributional islands are evident in *M. megacephalus*: western clade, Idaho isolate, main central unit, Mono isolate, and eastern clade (Table 4; Fig. 2). In addition to being geographically distinct from one another, these five distributional islands are genetically distinct (no haplotype sharing). Distributional islands vary in size from the tiny Idaho isolate (2.585 log_10_ km^2^) to the main central unit (4.937 log_10_ km^2^; Table 4). Comparing the number of unique composite haplotypes, *h*, with distributional island size for *M. megacephalus* (Table 4) yields a significant functional relationship (*b*=12.071, *P*=0.010; *r*^2^=0.920); genetic variation, as measured by the number of distinct haplotypes and area are directly related. When the analysis is expanded to include the four distributional islands of *M. pallidus* identified previously (see Hafner *et al.*, 2008; Table 4), we find a highly significant haplotype–area curve for *Microdipodops* (*b*=12.918, *P*<0.001; *r*^2^=0.922; Fig. 4).

**Table 4:** Distributional island area (Area in log_10_ km^2^), sample size (*n*), observed number of unique composite haplotypes (*h*), predicted number of haplotypes (*ĥ*), and probability of completeness (*P*) for the distributional islands of *Microdipodops* in the Great Basin Desert region of western North America. Names of distributional islands, *n*, and *h* for *M. pallidus* are taken from Hafner *et al.* (2008)

Distributional island	Area	*n*	*h*	*ĥ*	*P*
*Microdipodops megacephalus*					
Western clade	4.846	65	23	24	0.173
Idaho isolate	2.585	2	1	1	0.134
Central clade					
Main central unit	4.937	61	31	38	0.002
Mono isolate	3.449	8	8	Na*	Na*
Eastern clade	4.300	50	25	31	0.007
*Microdipodops pallidus*					
Western clade					
Main western unit	4.397	44	19	21	0.070
Deep Springs isolate	2.837	10	1	1	0.998
Eastern clade					
Main Eastern unit	4.125	41	21	26	0.011
Alamo isolate	3.090	3	1	1	0.609

* Analysis of completeness of haplotype sampling requires that *n* must be greater than *h*.

Comparison between the actual number of haplotypes recorded on a distributional island, *h*, and the predicted number, *ĥ*, reveals that sampling was generally thorough and sufficient to produce reliable assessments of genetic variation (Table 4). For most distributional islands there is remarkable agreement between observed and predicted number of haplotypes (Table 4). The three instances where the probability of completeness is significant (the main central unit and the eastern clade of *M. megacephalus* and the main eastern unit of *M. pallidus*) pertain to large distributional islands having the three highest numbers of predicted haplotypes (i.e. 38, 31 and 26, respectively; Table 4).

## Discussion

### Phylogenetic patterns and historical biogeography

The four principal clades identified in this study are distributed in an allopatric fashion with no known areas of sympatry (Fig. 2). Whereas most of the phylogroups are separated from one another by more than 100 km of unsuitable habitat, the central and eastern clades approach each other in a near-parapatric (contiguously allopatric) manner. Kangaroo mice belonging to the central and eastern clades are found nearest (*c.* 25 km) each other in White River Valley and Cave Valley (localities Sunnyside and Pony Springs, respectively; Figs 1 & 2). Preliminary fieldwork in this region shows that the intervening habitat is inappropriate for kangaroo mice. The only known area of sympatry involving *M. megacephalus* and other clades of kangaroo mice observed in this study occurs in the southern portion of the distribution of the central clade. Here, *M. megacephalus* is found sympatric with the eastern clade of *M. pallidus* (Hafner *et al.*, 2008).

Cladogenesis within *M. megacephalus* may be placed in a temporal framework of evolutionary divergence within the family Heteromyidae (Hafner *et al.*, 2007) and compared with diversification in *M. pallidus* (Hafner *et al.*, 2008). In general, the estimated divergence times from the beast analyses using the relaxed clock (UCLN) model appear older and have larger error intervals than do dates estimated using a strict clock (CLOC; Table 2). Since the UCLN model does not require molecular evolutionary rates to be inherited from node to node throughout the phylogeny, lineage-specific rate heterogeneity is allowed. It appears that decoupling rates among lineages in the UCLN model allows fast-evolving lineages to be older but with less certainty in their temporal placement. However, we interpret the CLOC model divergence-time estimates with higher confidence for several reasons, including the initial failure of the log-likelihood ratio test to reject the molecular clock, the greater specificity of CLOC error estimates, and the greater congruence between CLOC age estimates and Hafner *et al.*'s (2007) independent assessment of divergence times. Accordingly, our results suggest a middle Miocene (*c*. 14–15 Ma) split between *Dipodomys* and *Microdipodops*, a late Miocene (*c*. 7–8 Ma) divergence of the *M. pallidus* and *M. megacephalus* lineages, and a middle Pliocene divergence (*c*. 2–4 Ma) of the principal clades within *M. megacephalu*s. The timing of major branching events within *M. megacephalus* is generally synchronous with the divergence (4.38 Ma) of the eastern and western phylogroups of *M. pallidus* reported by Hafner *et al.* (2008).

Accumulating evidence from both molecular (Hafner *et al.*, 2006, 2007, 2008; this paper) and palaeontological (Remeika *et al.*, 1995; Cassiliano, 1999; Jefferson & Lindsay, 2006) studies suggest that kangaroo mice are a relatively old group that diverged during the Miocene and Pliocene and south of the Great Basin. Being sand-obligate mammals, kangaroo mice probably invaded the Great Basin following the formation of extensive sandy habitats during the Pleistocene pluvial–interpluvial cycles (Morrison, 1964; Smith, 1982; Mehringer, 1986; Eissmann, 1990). At this time, however, we cannot rule out the existence of sandy habitats suitable for kangaroo mice in the Great Basin during the Pliocene, owing to an ongoing dispute over the age of the Sierra Nevada uplift and the formation of the eastern rain shadow (dates range from Eocene to late Miocene or early Pliocene; for a review see Molnar, 2010). Although it appears likely that kangaroo mice are relatively recent invaders of the Great Basin (i.e. allochthonous endemics), this designation ultimately awaits more conclusive evidence of regional climatic and tectonic history. These inferences are in sharp contrast to previous interpretations of a relatively young genus that diverged recently and *in situ* in the Great Basin (Hall, 1941; Hafner, 1978).

### Comparisons with previous assessments

With a much smaller data set relative to this study, Hafner's (1981) isozymic data recognized three assemblages (the Idaho isolate, and main western and eastern units) that are generally consistent with the present findings based on mtDNA data. Specifically, Hafner's (1981) western and eastern units are consistent with the western and the central plus eastern clades, respectively, that are identified in this study. As in this study, Hafner (1981) and Hafner *et al.* (2006) showed that the kangaroo mice from the Mono peripheral isolate share ancestry with kangaroo mice from central Nevada.

Chromosomal data from Hafner (1981) provide additional nuclear corroboration of the general mtDNA patterns described here. Two karyotypes occur in *M. megacephalus* (Hafner, 1981): the 40-α karyotype (2*n* = 40, one pair of tiny acrocentric autosomes) and the 40-β karyotype (2*n* = 40, all bi-armed autosomes). The distributions of the 40-α and 40-β karyotypes from Hafner's (1981) eastern unit agree with the distributions of the central and the eastern clades, respectively. The Idaho isolate is characterised by the 40-β karyotype (Hafner, 1981, 1985) and, although this is not a unique karyotype, the distributional pattern of this karyotype is distinctive in that the nearest surrounding populations of kangaroo mice show the 40-α karyotype (Hafner, 1981). Hafner's (1981) western unit (=western clade from this study) shows both karyotypes; unfortunately, lack of phylogenetic resolution within the western clade prevents a comparison with the distribution of the karyotypes reported by Hafner (1981).

Hall (1941) recognized 12 subspecies of *M. megacephalus* based on his examination of cranial and external morphological characters. There appears to be no correspondence between the phylogenetic patterns outlined here and the patterns of phenetic variation summarized for *M. megacephalus* by Hall (1941). This discordance is somewhat surprising when compared with the general agreement found between phylogeographical patterns (Hafner *et al.*, 2008) and subspecies distributions (Hall, 1941) in *M. pallidus*. Given that the distribution of *M. megacephalus* is *c*. 4.5 times larger and encounters a wider range of environmental conditions than that of *M. pallidus*, the phenetic patterns identified by Hall (1941) for *M. megacephalus* most likely reflect mainly adaptive modifications rather than components of shared ancestry.

### Cryptic speciation

Sequence divergence in cytochrome *b* is now recognized as the ‘industry standard’ for assessing molecular divergence in phylogenetic studies (Meyer, 1994, p. 278). Mean pairwise sequence-divergence values for cyt *b* between the four principal phylogroups of *M. megacephalus* are 7.89% and 8.54% for uncorrected *p* and Kimura's two-parameter model, respectively (Table 1). As mentioned by Hafner *et al.* (2008), these values of cyt *b* sequence-divergence should be regarded as conservative estimates because they are based on examination of the first portion of the gene, which is known to contain a functioning redox centre in the electron transport chain (Howell, 1989; Irwin *et al.*, 1991) and evolves at a slower rate than the second portion of the gene in rodents and other mammals (Irwin *et al.*, 1991; Lara *et al.*, 1996; Spotorno *et al.*, 2004). Despite the conservative nature of these sequence-divergence values, the level of differentiation of the phylogroups of *M. megacephalus* is consistent with mean percentage sequence-divergence values (> 5%) often reported for sister species of mammals (Baker & Bradley, 2006). Hence, the four major phylogroups identified here are likely to be genetically isolated species.

The four main phylogroups appear to represent morphologically cryptic species embedded within the taxon, *M. megacephalus*. Given that the two basal clades of kangaroo mice, *M. megacephalus* and *M. pallidus*, are regarded morphologically as sibling species (e.g. Hafner *et al.*, 1979), it is not surprising that we know of no morphological characters at this time that will permit discrimination among the major clades of *M. megacephalus*. Before these four major phylogroups are recognized taxonomically, additional research is warranted. Specifically, it would be useful to incorporate additional nuclear markers (e.g. further karyological analyses and especially the use of nuclear sequence data) to evaluate our phylogenetic patterns based on mtDNA data. Although the four clades appear to be distributed strictly in an allopatric fashion, additional reconnaissance in central Nevada (the area where the distributions of the central and eastern clades approach one another) would be valuable in determining whether the forms come into contact and, if so, the nature of the genetic interactions between them.

Many authors have noted that the dramatic climatic events of the Pleistocene were critical to the formation of the Great Basin's flora and fauna (e.g. Grayson, 1993). When considering the evolution and historical biogeography of a sand-obligate endemic such as kangaroo mice, it is especially attractive to focus on the Pleistocene's pluvial history and the formation of sandy habitats as key elements facilitating adaptive divergence. However, evidence from this study and Hafner *et al.* (2008) indicates that major lineage divergence within *Microdipodops* pre-dated the tumultuous climatic events of the Pleistocene. It is unknown exactly how the pluvial events affected the distribution, abundance and divergence of kangaroo mice. We do note, however, that two of the four lineages in *M. megacephalus*, the western clade and the eastern clade, are distributed in the general vicinity of the two largest pluvial lakes of the Pleistocene (Lahontan and Bonneville, respectively) and seem to occur primarily in fine sands in lower elevational habitats. The other two clades of *M. megacephalus*, the central clade and the Idaho isolate, occur in the central and northern Great Basin and are typically found on sandy soils with a gravel overlay, in middle-to-upper elevational habitats. From a historical–biogeographical perspective, it appears that multiple lineages of kangaroo mice invaded the Great Basin perhaps in the early Pleistocene; two major lineages of *M. pallidus* (Hafner *et al.,* 2008) and four major lineages of *M. megacephalus* survive today as products of cryptic speciation.

### Intrapopulational haplotypic variation in kangaroo mice

Direct comparisons of sequence data for *M. megacephalus* (this study) and *M. pallidus* (Hafner *et al.*, 2008) are possible because both studies relied on the same gene fragments. Inferences concerning intrapopulational haplotypic variation may be made by examining those general localities where multiple individuals were examined (38 and 20 localities for *M. megacephalus* and *M. pallidus*, respectively); there is no significant difference between the mean number of individuals sampled per locality for the taxa (means are 4.66 and 4.45 for *M. megacephalus* and *M. pallidus*, respectively; *U*=348.500, *P*=0.598). Comparisons between *M. megacephalus* and *M.* pallidus regarding 16S show no significant difference for mean number of haplotypes (2.05 and 1.95, respectively; *U*=416.5, *P*=0.523) nor for mean number of polymorphic sites per locality (2.05 and 1.40, respectively; *U*=432.0, *P*=0.380). However, comparisons between *M. megacephalus* and *M. pallidus* for cyt *b* show a marginally significant difference between the mean number of haplotypes (2.50 and 1.90, respectively; *U*=490.5, *P*=0.054) and a strongly significant difference between mean number of polymorphic sites per locality (3.89 and 1.20, respectively; *U*=543.0, *P*=0.007).

Intrapopulational genetic differences between *M. megacephalus* and *M. pallidus* should be more pronounced and more easily detected statistically in cyt *b* than in 16S owing to the higher rate of substitution in cyt *b*. Higher levels of population genetic variability in *M. megacephalus* relative to *M. pallidus* may relate to the fact that this species has a larger distribution, a morphology that appears to be more generalized and variable, and inhabits a wider variety of edaphic and floral conditions than *M. pallidus* (Hall, 1941; Hafner, 1981; Hafner *et al.*, 2008). Although it may be tempting to invoke natural selection and Van Valen's (1965) niche-variation hypothesis to explain the observed higher levels of within-populational haplotypic variability in cyt *b* for *M. megacephalus*, it is more parsimonious to conclude that the mechanisms responsible for the observed differences are mainly mutation and genetic drift (and not selection). As noted earlier, cyt *b* for kangaroo mice evolves largely in a neutral fashion (the McDonald–Kreitman test for selective neutrality was not significant).

Given their differences in geographical distributions and habitat preferences, it is likely that *M. megacephalus* and *M. pallidus* experienced dissimilar histories of genetic bottlenecks. Without doubt, the sizes and numbers of populations of kangaroo mice have fluctuated through time in response to environmental changes and populations have lost haplotypic variation due to genetic drift. Greater mean haplotypic variation in cyt *b* for populations of *M. megacephalus* suggests that *M. megacephalus* may have realized larger average population sizes over time than *M. pallidus* (although we found no significant difference between the mean number of individuals sampled per locality between the taxa) and/or *M. megacephalus* may have endured less recent and less severe bottlenecks than *M. pallidus*. Hopefully, future work on the population genetics of kangaroo mice and more detailed information regarding past climatic changes in the Great Basin will enable an evaluation of the demographical histories of these forms.

### Directional analyses and source–sink dynamics

Analyses of axial data pertaining to haplotype sharing patterns over geography reveal signatures of historical routes of gene exchange when evaluated by DAPP (Hafner *et al.*, 2008). The two statistically significant orientation patterns uncovered in this study (a north–south directional trend and the web pattern; Fig. 3b) suggest that populations of kangaroo mice adjusted their distributions in response to past climatic changes such as those during the Pleistocene. Specifically, the north–south angular trends are indicative of climate-induced northward and southward distributional adjustments and the web pattern suggests that there was a refugium in the southern Great Basin during cooler climatic periods. Additionally, these angular trends may provide evidence for source–sink population structure (e.g. Pulliam, 1988; Dias, 1996) in kangaroo mice. Hence, there are two explanations for the angular trends that are not mutually exclusive. The age of these haplotype-sharing patterns is not known at this time but would be useful in evaluating these explanations. The web pattern shown here for *M. megacephalus* was not observed in the companion study of *M. pallidus* (Hafner *et al.*, 2008).

The majority (52 of 66 total pairwise combinations) of the axial data available for DAPP pertain to haplotype sharing in the central clade. The co-occurrence of the north–south and web angular trends in the central clade (Figs 2 & 3b) provides telltale signs of source–sink population dynamics. The central clade may be envisioned as a source–sink metapopulation composed of subpopulations of kangaroo mice inhabiting patches of suitable habitat. The northern-most subpopulations here (i.e. Contact, Cobre, Ruby Valley; Figs 1 & 3) contain kangaroo mice in low densities (mean number of animals collected per locality = 1.67) and exist in tiny, isolated patches; these may be regarded as sink subpopulations. Although systematic assessment of habitat quality was not made, these northern patches were judged by us to be low-quality habitats relative to more southern sites (Contact and Cobre had much gravel overlay and Ruby Valley had unusually tall vegetation). In contrast, the 11 southern subpopulations involved in the web pattern (formed by sharing of two haplotypes among six and seven localities each; Table 3) may be viewed as source subpopulations; kangaroo mice occur at slightly higher densities in these subpopulations (mean number of animals collected = 2.82) and in larger patches than the extreme northern subpopulations. In addition, these southern subpopulations are genetically more variable than the northern subpopulations (mean number of haplotypes is 2.09 and 1.33 in the southern and northern subpopulations, respectively), suggesting more long-term stability in the southern, source region. Given these characteristics, it is likely that these northern subpopulations are more prone to extinction and may be maintained by immigration from kangaroo mice in the southern source patches. It is noteworthy that the numbers of private haplotypes in the northern (sink) and southern (source) subpopulations are similarly low (zero or one private haplotype per locality; Fig. 3a) but this is likely to be for different reasons. The dearth of private haplotypes in the northern subpopulations is probably due to local extinction followed by recent colonization from the southern subpopulations, whereas the low number of private haplotypes in the southern subpopulations suggests high levels of gene exchange and a relatively stable demography over long periods of time.

Source–sink dynamics and metapopulation theory may provide a useful framework for future studies examining population regulation, demography, and conservation biology of kangaroo mice. We encourage future workers to incorporate source–sink theory and to gather data regarding the size and quality of a habitat patches. As Figueira & Crowder (2006) noted, source–sink theory has provided much assistance to conservation biologists and wildlife managers in identifying source and sink patches, population size, patch contribution, dispersal corridors, and metapopulation persistence.

### Distributional islands of kangaroo mice

As pointed out by MacArthur (1972, p. 105), ‘Some mainland habitats are obviously islands’. This perspective is especially relevant when considering the distribution of a stenotopic, mainland taxon such as *Microdipodops*. Given their sand-obligate ecology, local populations of kangaroo mice are distributed in a patchy manner across the Great Basin and are aggregated into distinct island populations that are defined genetically and geographically (Fig. 2; Hafner *et al.*, 2008).

Kangaroo mice inhabiting a distributional island may be viewed as an isolated population surrounded by an ecological vacuum or ‘sea’. Although the larger distributional islands are likely to represent metapopulations, the size of a distributional island serves as a correlate of overall population size (see also Frankham, 1996). As predicted by population genetics theory, there is a high positive correlation between genetic diversity and the size of distributional islands in kangaroo mice (Fig. 4). Population genetics theory predicts that genetic variation is a balance between mutation, drift, and natural selection in a population of finite size. There is now a growing body of empirical evidence that demonstrates the positive correlation between genetic variation and population size (and island size) both across populations of a species and across species of plants and animals (for review see, Soulé, 1976; Frankham, 1996).

The haplotype–area curve (Fig. 4) for the distributional islands of *Microdipodops* is analogous to the familiar species–area curves from the theory of island biogeography (MacArthur & Wilson, 1967; MacArthur, 1972), and it may be tempting to apply this theory to our data. However, here we are examining genetic diversity (number of unique haplotypes) at the populational level rather than species diversity (i.e. species richness) at the community level. Given that there is no sharing of composite haplotypes among any of the nine distributional islands, it is unnecessary to invoke a possible balance between immigration and extinction from island equilibrium theory. Instead, population genetics theory alone is sufficient to explain the haplotype–area curve (Fig. 4) for the distributional islands of *Microdipodops*.

Iguchi & Nishida (2000) performed a similar analysis of haplotypic diversity and island size in their study of an osmeroid fish in the Japanese Archipelago, yet we believe that application of this approach to a mainland taxon is both novel and useful in phylogeographical studies and we encourage its application in future studies. Understanding haplotypic variation in space and time is important in the context of conservation biology of kangaroo mice. The high correlation of the haplotype–area curve (Fig. 4) suggests that the populations of kangaroo mice represented by the distributional islands are now in approximate genetic equilibrium. As such, it appears that there have not been recent genetic bottlenecks for any of the larger distributional islands (i.e. for distributional islands equal to or larger than the Mono isolate; Table 4) that were sufficiently egregious to disrupt the formation of a functional trend. Given the great climatic fluctuations during the Pleistocene and the patchy distribution of the subpopulations, this finding was rather surprising to us. The highly significant trend implies that either population sizes for the larger distributional islands did not fluctuate wildly during the pluvial history of the Pleistocene or that genetic equilibrium formed since the end of the Pleistocene. It is noted, however, that these results from maternally inherited mtDNA are most accurately interpreted as dynamics of effective female population size through time. Nonetheless, the high number of unique composite haplotypes from the larger distributional islands indicates that source subpopulations probably persisted throughout the turbulent history of the Pleistocene and were sufficiently large to preserve and accumulate nucleotide substitutions over time; it is not known how long it took for equilibrium to be achieved. The high diversity of haplotypes recorded from the larger distributional islands appears promising for future conservation efforts but the lack of mtDNA variation on the three smallest distributional islets (Idaho, Deep Springs, and Alamo isolates; Fig. 4 & Table 4) is discouraging, albeit entirely predictable from traditional population genetics theory (e.g. Wright, 1931). All distributional islands of kangaroo mice show unique mtDNA properties, so the loss of any distributional island (small or large) would affect adversely overall kangaroo mouse genetic diversity. Future conservation efforts for *Microdipodops* should focus on ensuring the welfare of the smaller and more vulnerable distributional islets while simultaneously working to maintain the genetic diversity represented in the metapopulations inhabiting the larger distributional islands.

### Kangaroo mouse abundance and changing abundance

As noted by Hafner *et al.* (2008), the routine reporting of measures of relative abundance (e.g. percentage trap success or capture rate) in phylogeographical studies is useful to field biologists, conservationists and wildlife managers. Such data are invaluable in monitoring the viabilities of populations, especially for organisms such as kangaroo mice, which are considered rare in nature (Hall, 1941, 1946; Hafner *et al.*, 2008). *M. megacephalus* is legally protected in California and Nevada but not in Idaho, Oregon, and Utah. Based on fieldwork from the 1970s, Hafner & Hafner (1998, p. 79) reported that the conservation status (IUCN Red List Category) of *M. megacephalus* was ‘Lower Risk, least concern’ (updated category is now ‘Least Concern’; Linzey & Hammerson, 2008).

Assessing the conservation status of kangaroo mice using the criteria of Mace & Lande (1991) requires information pertaining to abundance and changing abundance over time. Our trapping results show that *M. megacephalus*, like *M. pallidus* (Hafner *et al.*, 2008), is among the least abundant of the nocturnal desert rodents in sandy habitats of the Great Basin. Considering only those localities where kangaroo mice were captured, the overall trapping success reported here for *M. megacephalus* (2.67%) is similar to the trapping success reported for *M. pallidus* (2.88%; Hafner *et al.*, 2008). When these trapping data for fieldwork conducted during 1999–2007 are combined, the overall *Microdipodops* trapping success is 3.03% (327 kangaroo mice captured/10,808 trapnights), mean percentage trap success is 3.43%, and mean number of kangaroo mice per site is 3.85 for 85 sites that yielded *Microdipodops*. These data may be compared directly with kangaroo mouse trapping data and fieldwork performed three decades ago (Hafner, 1981). Hafner's fieldwork during 1972–1979 showed overall *Microdipodops* trapping success was 4.32% (442 kangaroo mice captured/10,233 trapnights), mean percentage trap success was 5.24%, and mean number of kangaroo mice per site was 6.70 for 66 sites that yielded *Microdipodops* (Hafner, 1981; data available on request). Comparison of trapping data between the time periods shows statistical significance for both mean percentage trap success (*U*=3377.5, *P*=0.031) and for mean number of kangaroo mice per site (*U*=3355.5, *P*=0.037). Kangaroo mice, long considered as rare by naturalists, now appear to be even less abundant.

### Habitat affinity

Fieldwork shows that *M. megacephalus* occurs in the upper portion of the Upper Sonoran Life-Zone and is found in habitats that are characterized by sandy soils (with or without a gravel overlay) and dominated by sagebrush, *Artemisia* Linnaeus and/or rabbit brush, *Chrysothamnus* Nuttall. Aside from an anomalous high-elevational record of 2455 m (8050 ft; Egoscue, 1981; see below), all capture records of *M. megacephalus* occur from 1189 m (3900 ft; Smoke Creek, Nevada; Hall, 1941) to 2164 m (7100 ft; 2.5 miles NW Powell Mountain, Nevada; Hafner *et al.*, 2006). Hall's (1941) report of two specimens taken at 2316 m (7600 ft) in Monitor Valley near our Belmont locality is erroneous; Monitor Valley does not exceed 2134 m (7000 ft) in this region. Elevationally, *M. megacephalus* occurs typically in sandy habitats below the singleleaf pinyon, *Pinus monophylla* Torrey & Frémont, and juniper, *Juniperus* Linnaeus, association and above those habitats where greasewood, *Sarcobatus* Nees von Esenbeck, and saltbush, *Atriplex* Linnaeus, predominate. At its lowest elevational and floral limits (e.g. Smoke Creek, Valley Falls, Fields and Panaca), *M. megacephalus* is found in very sandy habitats dominated by greasewood and/or saltbush and often with rabbit brush present. The habitats harbouring *M. megacephalus* at its upper elevational and floral limits (e.g. Powell Mountain, Belmont and Cobre) are dominated by sagebrush and in sandy soils with a gravel overlay immediately below the pinyon–juniper belt. Egoscue's (1981) unusual high-elevational record pertains to kangaroo mice caught in pinyon–juniper habitat near the summit of a mountain pass during the post-reproductive period and probably represents the fortuitous capture of dispersing individuals.

Throughout its distribution, *M. megacephalus* occurs in a variety of floral associations and, although restricted to sand, displays a rather broad tolerance for soils with varying amounts of gravel overlay. In contrast to *M. megacephalus*, *M. pallidus* is usually found in habitats above those that support the creosote bush, *Larrea* Cavanilles, and below those that support sagebrush (Hafner *et al.*, 1996, 2008). *M. pallidus* is found most frequently in deep, fine, sandy soils and in floral communities where greasewood and saltbush predominate; such habitats occur in the lower portion of the Upper Sonoran Life-Zone. Future studies examining the ecology and habitat specificity of kangaroo mice may find it fruitful to examine possible differences among the principal clades of *M. megacephalus* recognized in this study (Fig. 2). Specifically, it would be interesting to know if the genetic divergence detailed here is accompanied by ecological specialization.

### Distribution and conservation biology

Our portrayal of the geographical range of *M. megacephalus* (Fig. 1) is similar to Hall's (1941) distribution map, excepting for the range extensions in Idaho and Utah and alterations of the distribution around the southern end of Pyramid Lake. It is important to bear in mind that the distribution depicted in Fig. 1 reflects all populations of *M. megacephalus* sampled from the wild in the course of this study and augmented by older specimens from key localities where trapping efforts during this study were unsuccessful. Localities of particular relevance here are Powell Butte, Riddle and Callao (Fig. 1); these localities are positioned on the northern periphery of the distribution and are represented by specimens collected more than 30 years ago. More information is needed on the status and conservation biology of these northern populations before definitive statements regarding temporal distributional adjustments can be made.

Despite recent concerns regarding global warming and documented changes in species distributions (e.g. Parmesan *et al.*, 1999; Beever *et al.*, 2003; Parmesan & Yohe, 2003; Wagner *et al.*, 2003; Perry *et al.*, 2005), we note no overall pattern of northward or elevationally upward distributional changes for *M. megacephalus* when comparing our capture data with those obtained three-quarters of a century ago by Hall (1941). These findings agree with those for *M. pallidus* (Hafner *et al.*, 2008) and are consistent with those reported for xeric-adapted species of mammals (including *M. megacephalus*) from north-eastern Nevada (Rowe *et al.*, 2010). A general pattern that emerged from our fieldwork, however, was the surprising and rather consistent difficulty of collecting specimens across the northern portions of the distribution. Many northern localities (e.g. Powell Butte, Narrows, Riddle, Quinn River Crossing, Sulphur, Winnemucca, Golconda, Izenhood, Halleck, and Callao) that were sampled successfully by Hall (1941) and/or by Hafner (1981) yielded no kangaroo mice in the course of our fieldwork. Other northern localities often yielded kangaroo mice in low abundance (only one or two specimens; e.g. Ruby Valley, Contact, Cobre, Cherry Creek). An exception to this pattern is Valley Falls: one kangaroo mouse was captured from 340 trapnights in 1978 but 13 kangaroo mice were taken from 400 trapnights in 2004.

Relative to the southern portions of their distribution, populations of kangaroo mice from the northern portion of the geographical range seem to show low abundance, occur in tiny habitat patches, and are more widely separated from each other. It is also evident from fieldwork over the past 30 years that many populations in the northern portion of the distribution of *M. megacephalus* have suffered severe habitat alteration and loss. Of the possible ‘big four’ threat factors discussed by Hafner *et al.* (2008), wild fires and invasive plants, especially over the past two decades, have devastated the low-elevational habitats across the northern portions of the Great Basin. Wild fires followed by the immediate invasion of introduced annual grasses and weed species (especially cheat grass, *Bromus tectorum* Linnaeus, and Russian thistle, *Salsola tragus* Linnaeus; Whisenant, 1990; Knapp, 1996) appear directly responsible for our inability to collect kangaroo mice at localities such as Winnemucca, Izenhood and Halleck (type locality for both the genus and species, *Microdipodops megacephalus*). Other kangaroo-mouse localities of Hall (1941) and Hafner (1981) not ravaged by fire are now modified to varying degrees by the presence of introduced annual grasses. Although it remains to be determined to what extent kangaroo mice can tolerate invasive plants, places that yielded kangaroo mice in the 1970s (e.g. Narrows, Quinn River Crossing and Sulphur) are now covered by invasive grasses and our collecting efforts yielded no kangaroo mice.

The most northern record for the genus is Powell Butte (Hall, 1941; Fig. 1, Appendix S1) and is based on a single specimen collected in 1920. This locality is over 150 km north of the closest known locality of kangaroo mice (Valley Falls; Fig. 1) and, because of its unique location, may provide insights into the conservation biology of kangaroo mice occurring at their upper ecological limits. Although our trapping efforts yielded no additional kangaroo mice, this locality appeared to represent satisfactory *Microdipodops* habitat except for the presence of juniper woodland. Because kangaroo mice occur below the limits of the juniper woodland elsewhere in their distribution, we conclude that woodland expansion, commonplace across the northern Great Basin since post settlement times due largely to fire suppression (Tausch *et al.*, 1981; Miller & Rose, 1999; Miller & Tausch, 2001), has resulted in the dissection and loss of sagebrush habitat and the extinction of this isolated population of kangaroo mice.

More so than in any other area across the distribution of *Microdipodops*, many populations of *M. megacephalus* in the northern portion of the Great Basin are either locally extinct or facing serious threats due to loss of habitat. Although some of the northern-most localities still seem to remain in a near-pristine ecological state (e.g. Valley Falls, Riddle, Fields, Contact, Cobre and Callao), their preservation is only due to happenstance of their extreme remote locations away from human settlements and activities. It should also be kept in mind that these populations are typically small and isolated (owing to the vagaries of the distribution of appropriate sandy habitats) and, hence, are highly susceptible to habitat alteration due to anthropogenic factors and the vicissitudes of climate change. From a conservation perspective, the picture that is emerging for *M. megacephalus* is one that parallels closely the environmental threats facing the sage grouse, *Centrocercus urophasianus* Bonaparte, in the Great Basin (e.g. Connelly *et al.*, 2004). Specifically, populations in the basins and valleys (towards their lower ecological range) are facing ever-increasing environmental threats and habitat loss due to wild fires, invasive plants, agriculture and livestock grazing, whereas populations of kangaroo mice occurring at higher elevations and in more northern latitudes (towards their upper ecological range) seem to be facing increasing loss of sagebrush habitat associated with expansion of juniper and pinyon woodland. Further fieldwork in these northern areas would be especially useful for monitoring the status and understanding the temporal stability of these small and isolated populations.

## Biosketches

**John C. Hafner** is Director and Curator of Birds and Mammals and Professor of Biology, Emeritus, and held a joint appointment in the Moore Laboratory of Zoology and the Department of Biology at Occidental College for 28 years. He continues his research in retirement and his interests focus on bird and mammal evolutionary biology, particularly the systematics and macroevolution of New World rodents of the superfamily Geomyoidea.

**Nathan S. Upham** is currently a doctoral student at the University of Chicago with research interests centred on the ecological and biogeographical aspects of diversification in mammals, especially caviomorph rodents in the superfamily Octodontoidea. He recently completed his Master's degree at Occidental College, where he studied the influence of moonlight on the activity patterns of nocturnal desert rodents in the Great Basin.

## SUPPORTING INFORMATION

**Appendix S1** Localities and numbers of specimens examined in this study.

**Appendix S2** Primers used for the amplification and sequencing of mitochondrial genes from *Microdipodops megacephalus* for ancient DNA analyses.

As a service to our authors and readers, this journal provides supporting information supplied by the authors. Such materials are peer-reviewed and may be re-organized for online delivery, but are not copy-edited or typeset. Technical support issues arising from supporting information (other than missing files) should be addressed to the authors.
