# Supplementary material for: Phylogeography of the dark kangaroo mouse, Microdipodops megacephalus: cryptic lineages and dispersal routes in North America's Great Basin
Source: J Biogeogr. 2011 Jun;38(6):1077–97. doi: 10.1111/j.1365-2699.2010.02472.x (PMC3151553; doi:10.1111/j.1365-2699.2010.02472.x)
Supplement: Supplementary file 1 [file jbi0038-1077-SD1.doc]

**SUPPORTING INFORMATION**

**Phylogeography of the dark kangaroo mouse, *Microdipodops megacephalus*: cryptic lineages and dispersal routes in North America’s Great Basin**

John C. Hafner and Nathan S. Upham

*Journal of Biogeography*

# APPENDIX S1

Localities and numbers of specimens of *Microdipodops megacephalus, M. pallidus, Dipodomys deserti* and *D. microps* from the Great Basin Desert region of western North America examined in this study. Specimens are deposited in the Moore Laboratory of Zoology (MLZ, Occidental College), the Museum of Southwestern Biology (MSB, University of New Mexico), Monte L. Bean Life Science Museum (BYU, Brigham Young University), San Diego Natural History Museum (SDNHM), Idaho Museum of Natural History (IMNH, Idaho State University), and the Museum of Vertebrate Zoology (MVZ, University of California, Berkeley). General localities for *M. megacephalus* are shown in bold and are listed alphabetically (ignoring compass-direction modifier); general localities are shown in Fig. 1.

*Microdipodops megacephalus* (*n* = 186). **AUSTIN**: 6.2 miles S, 19.6 miles W Austin, 6150 feet, Lander County, Nevada (*n* = 4, MLZ 1748-1751). **BELMONT**: 3.2 miles N, 4.2 miles E Belmont, 7000 feet, Nye County, Nevada (*n* = 4, MLZ 2027-2030). **BENTON**: 5 miles N Benton, 5600 feet, Mono County, California (*n* = 6, MLZ 1740-1742, MLZ 1915-1917). **BERYL**: 0.7 miles N, 6.3 miles E Beryl, 5125 feet, Iron County, Utah (*n* = 8, MLZ 2145-2152). **CALLAO**: 7.7 miles S, 2.7 miles E Callao, 4500 feet, Juab County, Utah (*n* = 2, MSB 35599, 35600); 5.5 miles S, 7.8 miles E Callao, 4400 feet, Juab County, Utah (*n* = 1, MSB 35602). **CHERRY CREEK**: 7.2 miles N, 8.8 miles E Cherry Creek, 5850 feet, White Pine County, Nevada (*n* = 1, MLZ 1965). **CHILCOOT**: 1.7 miles N Chilcoot, 5100 feet, Plumas County, California (*n* = 1, MLZ 1756); 1.5 miles N Chilcoot, Plumas County, California (*n* = 1, MVZ 158930). **COBRE**: 0.9 miles S, 0.4 miles W Cobre, 5900 feet, Elko County, Nevada (*n* = 2, MLZ 2067, 2068). **CONTACT**: 10.9 miles S, 2.5 miles W Contact, 5700 feet, Elko County, Nevada (*n* = 2, MLZ 2069, 2070). **CURRANT**: 4.9 miles S, 28.2 miles W Currant, 6000 feet, Nye County, Nevada (*n* = 2, MLZ 2005, 2006). **DANVILLE**: 6.1 miles S, 2.4 miles E Danville, 6800 feet, Nye County, Nevada (*n* = 3, MLZ 2021-2023). **DENIO**: 0.6 miles S Denio, 4200 feet, Humboldt County, Nevada (*n* = 2, MSB 35530, 35531). **DUCKWATER**: 8.4 miles N, 17.5 miles W Duckwater, 6350 feet, Nye County, Nevada (*n* = 3, MLZ 1997-1999). **N EUREKA**: 22.8 miles N, 3.6 miles W Eureka, 5850 feet, Eureka County, Nevada (*n* = 4, MLZ 1956, 1957, MSB 35526, 35527). **W EUREKA**: 6.2 miles N, 9.5 miles W Eureka, 6000 feet, Eureka County, Nevada (*n* = 2, MLZ 2031, 2032). **FIELDS**: 2.4 miles N, 3.4 miles E Fields, 4050 feet, Harney County, Oregon (*n* = 9, MLZ 2007-2015). **FLETCHER**: ¼ mile N Fletcher, 6100 feet, Mineral County, Nevada (*n* = 2, MLZ 1744, 1745). **GERLACH**: 28.5 miles N, 27.8 miles W Gerlach, 4700 feet, Washoe County, Nevada (*n* = 5, MLZ 2089-2093); 28.2 miles N, 27.6 miles W Gerlach, 4700 feet, Washoe County, Nevada (*n* = 5, MLZ 2094-2098); 24.5 miles N, 25.0 miles W Gerlach, 4800 feet, Washoe County, Nevada (*n* = 1, MLZ 2099); 24.0 miles N, 24.8 miles W Gerlach, 4800 feet, Washoe County, Nevada (*n* = 5, MLZ 2100-2104); 22.4 miles N, 23.6 miles W Gerlach, 4800 feet, Washoe County, Nevada (*n* = 5, MLZ 2105-2109). **GEYSER**: 5.3 miles S, 1.6 miles E Geyser, 5900 feet, Lincoln County, Nevada (*n* = 2, MLZ 1974, 1975); 5.2 miles S, 1.9 miles E Geyser, 5900 feet, Lincoln County, Nevada (*n* = 4, MLZ 1976-1979); 5.1 miles S, 2.3 miles E Geyser, 5900 feet, Lincoln County, Nevada (*n* = 4, MLZ 1980-1983). **GOLDFIELD**: 12.0 miles N, 2.5 miles W Goldfield, 4860 feet, Esmeralda County, Nevada (*n* = 1, MLZ 1747). **GOLD REED**: 2.9 miles S, 3.1 miles E Gold Reed, 5350 feet, Nye County, Nevada (*n* = 1, MLZ 2053); 2.9 miles S, 4.0 miles E Gold Reed, 5330 feet, Nye County, Nevada (*n* = 5, MLZ 2054-2058). **N HIKO**: 31 miles N, 1 mile W Hiko, 5100 feet, Lincoln County, Nevada (*n* = 1, MLZ 1960). **W HIKO**: 6 miles N, 31 miles W Hiko, 4800 feet, Lincoln County, Nevada (*n* = 2, MLZ 1815, 1816). **JUNGO**: 13.8 miles N, 11.2 miles E Jungo, 4200 feet, Humboldt County, Nevada (*n* = 5, MLZ 2124-2128). **MILFORD**: 16.1 miles S, 19.6 miles E Garrison, 5400 feet, Millard County, Utah (*n* = 3, MLZ 2079-2081); 19.3 miles S, 18.4 miles E Garrison, 5100 feet, Millard County, Utah (*n* = 6, MLZ 2082-2087); 11.2 miles N, 39.6 miles W Milford, 5200 feet, Beaver County, Utah (*n* = 1, MLZ 2088). **MINERSVILLE**: 4.2 miles S, 15.8 miles W Minersville, 5050 feet, Beaver County, Utah (*n* = 8, MLZ 2071-2078); Escalante Desert, 38˚ 09.118’ N, 113˚ 12.946’ W, 1,540 m, Beaver County, Utah (*n* = 2, BYU 30100, 30101). **OSCEOLA**: 6.0 miles S, 4.2 miles W Osceola, 5800 feet, White Pine County, Nevada (*n* = 3, MLZ 1942-1944). **PANACA**: 24 miles W Panaca, 4600 feet, Lincoln County, Nevada (*n* = 4, MLZ 1752-1755). **PONY SPRINGS**: 9.0 miles N, 10.8 miles W Pony Springs, 6020 feet, Lincoln County, Nevada (*n* = 2, MLZ 2059, 2060). **POWELL BUTTE**: Becker Ranch, Powell Butte, Crook County, Oregon (*n* = 1, SDNHM 16431). **RAVENDALE**: 4.4 miles N, 13.6 miles E Ravendale, 5650 feet, Lassen County, California (*n* = 3, MLZ 2110-2112); 4.7 miles N, 10.8 miles E Ravendale, 5350 feet, Lassen County, California (*n* = 2, MLZ 2113, 2114). **RIDDLE**: Starr Valley, NW ¼ Section 19, T16S, R5W, B.M., Owyhee County, Idaho (*n* = 1, IMNH 259); ½ mile N Nevada, 2 ½ miles E Oregon, Owyhee County, Idaho (*n* = 1, IMNH 693). Note that the locality descriptor, “RIDDLE”, is taken from the type locality of *M. m. atrirelictus* (11 miles N, 44.2 miles W Riddle, 4200 feet, Owyhee County, Idaho) that is in the same approximate location as the aforementioned localities. **RUBY VALLEY**: 13.2 miles S, 0.6 miles E Ruby Valley, 6000 feet, Elko County, Nevada (*n* = 1, MLZ 2033). **SAN ANTONIO**: 3.7 miles N, 3.2 miles E San Antonio, 5600 feet, Nye County, Nevada (*n* = 2, MLZ 1761, 1762). **SPARKS**: 6 miles N, 4 miles E Sparks, 4600 feet, Washoe County, Nevada (*n* = 3, MLZ 1757-1759). **SUNNYSIDE**: 1.3 miles S, 4.9 miles W Sunnyside, 5200 feet, Nye County, Nevada (*n* = 1, MLZ 1966). **NE TONOPAH**: 13.8 miles N, 7.9 miles E Tonopah, 5800 feet, Nye County, Nevada (*n* = 4, MLZ 1961-1964). **SE TONOPAH**: 9.8 miles S, 9.9 miles E Tonopah, 5200 feet, Nye County, Nevada (*n* = 1, MLZ 1831). **TYBO**: 1.0 miles N, 8.5 miles W Tybo, 6200 feet, Nye County, Nevada (*n* = 2, MLZ 1799, 1800). **VALLEY FALLS**: 36 miles N, 14 miles E Valley Falls, 4300 feet, Lake County, Oregon (*n* = 10, MLZ 1987-1996). **VERNON**: 0.5 miles S, 11.5 miles W Vernon, 4450 feet, Pershing County, Nevada (*n* = 1, MLZ 1760). **VYA**: 3.2 miles N, 11.5 miles E Vya, 5600 feet, Washoe County, Nevada (*n* = 3, MLZ 1984-1986). **WARM SPRINGS**: 5.9 miles N, 10.2 miles E Warm Springs, 5200 feet, Nye County, Nevada (*n* = 1, MLZ 2024); 6.4 miles N, 10.1 miles E Warm Springs, 5200 feet, Nye County, Nevada (*n* = 1, MLZ 2025); 7.7 miles N, 9.5 miles E Warm Springs, 5200 feet, Nye County, Nevada (*n* = 1, MLZ 2026). **NE WARM SPRINGS**: 19.2 miles N, 13.4 miles E Warm Springs, 6000 feet, Nye County, Nevada (*n* = 5, MLZ 1905, MLZ 1948-1951). **SE WARM SPRINGS**: 12.7 miles S, 0.4 miles E Warm Springs, 6000 feet, Nye County, Nevada (*n* = 5, MLZ 1968-1972). **N WINNEMUCCA**: 7 miles N Winnemucca, 4600 feet, Humboldt County, Nevada (*n* = 2, MSB 35533, 35534). **SW WINNEMUCCA**: 5.5 miles S, 9.2 miles W Winnemucca, 4300 feet, Humboldt County, Nevada (*n* = 1, MSB 35535).

*Microdipodops pallidus*. 1.8 miles S, 5.3 miles E Coaldale, 4797 feet, Esmeralda County, Nevada (*n* = 1, MLZ 1817); 11.0 miles S, 10.0 miles E Tonopah, 5200 feet, Nye County, Nevada (*n* = 1, MLZ 1823).

*Dipodomys deserti*. 10.7 miles S, 25.0 miles W Gerlach, 3950 feet, Washoe County, Nevada (*n* = 1, MLZ 2065).

*Dipodomys microps*. 6 miles N, 0.5 miles W Bishop, 4200 feet, Inyo County, California (*n* = 1, MLZ 1765).

**APPENDIX S2** Primers used for the amplification and sequencing of mitochondrial genes from *Microdipodops megacephalus* for ancient DNA analyses. The primer name denotes the DNA strand (L = light, H = heavy) and the position of the 3’ end of the oligonucleotide relative to the numbering system for the *Mus* mitochondrial genome (Bibb *et al.*, 1981).

Gene Primer name Sequence (5’ to 3’)

16S rRNA^1^:

L1950 5’-CGC CTG TTT ACC AAA AAC ATC AC

L1962 5’-CCA AAA ACA TCA CCT CTA GCA TAA C L2109 5’-GGG TTT AAC TGT CTC TTA CAT TCA ATC A L2208 5’-CTC TCA ATT CTT ACA GAC CAG CCT AAA L2173 5’-TAA GAC GAG AAG ACC CTA TGG AGC L2262 5’-AAT TTT GGT TGG GGT GAC CTC L2270 5’-GTT GGG GTG ACC TCG GAG CAT A L2293 5’-GCA TAA ACT AAC CTC CGA AAG ACT ATC A

H2153 5’-AGC TCC ATA GGG TCT TCT CGT C

H2244 5’-CGA GGT CAC CCC AAC CAA AA H2315 5’-CTG GGT CAA TGT TTA GAC TTG GGT A H2419 5’-GAT CCA ACA TCG AGG TCG TAA ACC

H2489 5’-CTC AGA TCA CGT AGG ACT TTA ATC G

cyt *b*^2^:

L14117 5’-CGA AGC TTG ATA TGA AAA ACC ATC GTT GTC L14198 5’-TTG TCA ACC ACG CAT TCA TCG AC L14349 5’-GCA TTC TCA TCC GTC ACA CAT ATC TGC C L14416 5’-GCC AAC GGA GCA TCA CTA TTC TTT ATC TG

H14323 5’-CGG CAG ATA TGT GTG ACG GAT GAG AAT G

H14376 5’-GCT CCG TTG GCA TGT ATA TTT CG

H14542 5’-GCA GCC CCT CAG AAT GAT ATT TGT CCT C

________________________________________________________________________

^1^ 16S primer pairs included: L1950 / H2153; L1950 / H2244; L1962 / H2419; L2109 / H2315; L2109 / H2244; L2173 / H2419; L2208 / H2419; L2262 / H2489; L2270 / H2419; L2293 / H2489

^2^ cyt *b* primer pairs included: L14117 / H14323; L14198 / H14323; L14198 / H14376; L14198 / H14542; L14349 / H14542; L14416 / H14542.

**REFERENCE**

Bibb, M.J., Van Etten, R.A., Wright, C.T., Walberg, M.W. & Clayton, D.A. (1981) Sequence and gene organization of mouse mitochondrial DNA. *Cell*, **26**, 167-180.
